# The McGurk Illusion: A Default Mechanism of the Auditory System

**DOI:** 10.3390/brainsci13030510

**Published:** 2023-03-19

**Authors:** Zunaira J. Iqbal, Antoine J. Shahin, Heather Bortfeld, Kristina C. Backer

**Affiliations:** 1 Department of Cognitive and Information Sciences, University of California, Merced, CA 95343, USA; 2 Health Sciences Research Institute, University of California, Merced, CA 95343, USA; 3 Department of Psychological Sciences, University of California, Merced, CA 95353, USA

**Keywords:** McGurk illusion, audiovisual integration, spoken language processing, visemes, phonetic encoding

## Abstract

Recent studies have questioned past conclusions regarding the mechanisms of the McGurk illusion, especially how McGurk susceptibility might inform our understanding of audiovisual (AV) integration. We previously proposed that the McGurk illusion is likely attributable to a default mechanism, whereby either the visual system, auditory system, or both default to specific phonemes—those implicated in the McGurk illusion. We hypothesized that the default mechanism occurs because visual stimuli with an indiscernible place of articulation (like those traditionally used in the McGurk illusion) lead to an ambiguous perceptual environment and thus a failure in AV integration. In the current study, we tested the default hypothesis as it pertains to the auditory system. Participants performed two tasks. One task was a typical McGurk illusion task, in which individuals listened to auditory-/ba/ paired with visual-/ga/ and judged what they heard. The second task was an auditory-only task, in which individuals transcribed trisyllabic words with a phoneme replaced by silence. We found that individuals’ transcription of missing phonemes often defaulted to ‘/d/t/th/’, the same phonemes often experienced during the McGurk illusion. Importantly, individuals’ default rate was positively correlated with their McGurk rate. We conclude that the McGurk illusion arises when people fail to integrate visual percepts with auditory percepts, due to visual ambiguity, thus leading the auditory system to default to phonemes often implicated in the McGurk illusion.

## 1. Introduction

The McGurk illusion [1] has been widely used as a model for audiovisual (AV) integration of spoken language [2,3,4]. In the McGurk illusion, individuals exposed to audio /ba/ or /pa/ paired with a silent video of /ga/ or /ka/, respectively, often report hearing /da/ or /ta/. Based on the assumption that AV integration and the McGurk illusion rely on the same underlying neural mechanism, researchers have conducted fMRI studies using the McGurk manipulation to understand where and how AV speech integration occurs. They found heightened activity in the superior temporal sulcus/gyrus (STS/G) during McGurk perception of incongruent AV speech relative to perception of congruent speech. The STS/G was thus identified as a hub for this multisensory fusion [2,4]. These findings were further corroborated via transcranial magnetic stimulation (TMS), whereupon stimulation (down regulation) of the STS with TMS (location identified via individual-specific fMRI) reduced individual susceptibility to the McGurk illusion [3]. In light of these and other findings, there is a widely held assumption that the McGurk illusion and AV integration are linked mechanistically, and the McGurk illusion is used as a conduit for understanding the neurophysiology of AV development [1,5,6], AV integration as a function of aging [7], and clinical deficits (e.g., schizophrenia, [8]).

However, recent accounts from independent labs have raised doubts about past models and their conclusions regarding the mechanisms giving rise to the McGurk illusion, as well as its suitability as an index of AV integration efficacy [9,10,11,12]. Van Engen and colleagues [11,12] questioned the appropriateness of the McGurk illusion as a source of information about AV integration. Van Engen et al. [12], for example, showed that individuals with enhanced McGurk illusion susceptibility did not possess an added advantage in sentence recognition in noise, raising questions about the relationship between the McGurk illusion and the well-established AV integration benefit in spoken language comprehension [13,14,15]. In their later study, van Engen et al. [11] argued that because the McGurk illusion rarely occurs naturally and is based on isolated syllables, it does not reflect real-life communication situations, thus making the McGurk illusion inappropriate to use as a procedural tool for understanding the mechanisms of AV integration in spoken language. 

Moreover—and despite extensive investigation—the neural mechanisms that give rise to the McGurk illusion itself are not well understood. For example, the McGurk illusion has been distinguished from—and at times conflated with—the visual dominance illusion [16,17,18,19], whereby individuals hear the visually conveyed phoneme when presented with an incongruent AV pair. An example of visual dominance is when exposure to audio-/ba/ paired with video-/fa/ leads to hearing /fa/, and vice versa—exposure to audio-/fa/ paired with video-/ba/ leads to hearing /ba/ [16,19]. Neurophysiologically, this visual dominance effect is evident through amplitude changes in the N1-P2 auditory evoked potentials (AEPs), including the observation that the N1 AEP amplitude shifts to reflect the listener’s illusory auditory perception instead of the actual auditory input [19]. It has been proposed that the McGurk illusion is a case of the visual dominance illusion [9,18], partly because the McGurk illusion is reinforced when the auditory input is weakened. For example, the McGurk illusion benefits from lower sound intensity and increased noise-level [18,20]. Alsius et al. [18] argue that the McGurk illusion arises due to an array of “weak” auditory consonants, such as /b/, which can be easily confused with other stop consonants. These researchers further posit that, because place of articulation is a weak acoustic feature [18,20], the McGurk illusion is driven by vision (i.e., watching a talker’s mouth movements) dominating the acoustic signal’s place of articulation cues; a conclusion also hypothesized in Gonzales et al. [9].

Our proposal is that the McGurk illusion occurs due to ambiguity in the specific test stimuli, thus leading to a failure in AV integration, which in turn causes sensory systems to default to specific perceptual representations. In a recent study by Gonzales et al. [9], we proposed that ambiguity associated with the visual stimuli due to indiscernible place of articulation (e.g., /g/, /k/, /y/), led perceivers to default to seeing ‘/d/t/th/’ and subsequently hearing ‘/d/t/th/’, consistent with a visual dominance account of the McGurk illusion. While the first experiment in that study supported such an account, we failed to replicate those findings in a second experiment, which featured a different talker. This lack of definitive findings motivated the current study. 

Herein, we build on Gonzales et al., and further test our “auditory default” hypothesis that during the McGurk illusion, perceptual defaulting to ‘/d/t/th/’ occurs in the auditory modality, and not in the visual modality. Participants performed two tasks. In the McGurk task, individuals saw visual-/ga/, while listening to audio-/ba/ and reported what they heard. The other task was an auditory-only task, in which individuals listened to trisyllabic words and pseudowords with one phoneme replaced by silence. We chose silence as a means to completely remove the phoneme from the acoustics and induce optimum perceptual ambiguity, a key factor in our theoretical framework. Individuals transcribed exactly what they heard as opposed to what they thought the original word/pseudoword was. 

We reasoned that AV incongruency creates an ambiguous perceptual/phonetic situation, one which prevents successful AV integration and thus, allows a default auditory phonetic representation to dominate perception. Thus, we hypothesized that if the McGurk illusion is due to perceptual defaulting within the auditory modality, then (1) individuals should default to (i.e., perceptually fill-in) ‘/d/t/th/’ (most weighted phonemes) for the silent gap in the auditory-only task; and (2) individuals with robust auditory-only ‘/d/t/th/’ default perception should exhibit stronger McGurk susceptibility with heightened ‘/d/t/th/’ perception. Addressing why both phenomena default to ‘d/t/’th/’ instead of other phonemes is beyond the scope of the present study, but we refer readers to Anderson et al., 2003 [21] for a possible argument. Finally, we used words and pseudowords to explore how lexical knowledge influences this auditory default processes. If the ‘/d/t/th/’ default (that is, the filling-in of missing auditory segments) is driven by prior lexical knowledge, then we should expect a higher ‘/d/t/th/’ default in the word condition. On the other hand, we should see more ‘/d/t/th/’ defaults in pseudowords if a ‘/d/t/th/’ default is driven by lexical ambiguity, since pseudowords are more ambiguous than words.

## 2. Materials and Methods

### 2.1. Participants

Thirty-seven young adults participated in this study. However, three participants were excluded from data analysis due to technical issues during data collection or not being a native/fluent English speaker, resulting in usable data from thirty-four participants (>18 years of age, M = 20.73 years, SD = 2.13 years, 2 participants did not provide their specific age; 27 females, 4 males, 3 did not respond; native or fluent English speakers). Of these thirty-four individuals, 29 reported that they are right-handed, 2 left-handed, and 3 ambidextrous. All participants self-reported normal hearing, normal or corrected vision, and no language deficits. Participants were recruited via an internal recruiting system of the University of California, Merced and provided written consent prior to participation. All experimental protocols were approved by the Institutional Review Board (IRB) of the University of California, Merced, and all methods were carried out in accordance with the guidelines and regulations of the IRB of the University of California, Merced and in accordance with the Declaration of Helsinki. Participants were monetarily compensated for their participation.

### 2.2. Stimuli

The study consisted of two tasks. The stimuli in one task consisted of English words and pseudowords spoken by a female talker (mean f0 = 203 Hz; see [22,23] for more details) with one phoneme replaced by silence. There was a total of 39 words and 36 pseudowords. Consonants were manually removed in Adobe Audition (Adobe Systems Inc., San Jose, CA, USA). For each word/pseudoword, one of the following consonants was removed from either the second or third syllable and replaced with silence: /k, t, d, g, b, ʃ, s, ʒ, z, ʧ, ʤ, l, r/. These phonemes were selected to ensure that there was a distribution of 2–4 consonants for each category of manner or place of articulation (i.e., stops, fricatives, bilabial, alveolar, etc.). Furthermore, the number of consonants removed from the second or third syllable was balanced across words and pseudowords; 15 consonants and 14 consonants were removed from the second syllable of words and pseudowords, respectively, and 24 and 22 consonants were removed from the third syllable of words and pseudowords, respectively. 

The other task involved a classic McGurk design whereby individuals listened to and watched a talker (two female talkers, mean f0 = 199 Hz, 184 Hz) uttering congruent and incongruent consonant vowels (CVs). The purpose of this task was to test the subjects for McGurk susceptibility. To create the stimuli, audio recordings of /ba/, /da/, and /ga/ were used along with video recordings of the talkers producing the same CVs. The videos were cropped, ensuring that participants could only view the space between the bridge of the talker’s nose and the bottom of the neck. This was to encourage participants to focus on the mouth and not be distracted by other features, such as the talker’s eyes. To create the AV pairings of congruent /ba/, congruent /da/, congruent /ga/, and incongruent /ba-ga/ (auditory /ba/ paired with visual /ga/ or viseme /ga/), the auditory stimuli of each talker were temporally aligned to the acoustic onset of the video, respectively. The temporal alignment included a natural auditory delay as is typical of natural utterances. This resulted in 32 stimuli (2 talkers × 4 AV pairings × 4 exemplars). The first half of participants were presented with stimuli of the first talker and the remaining participants were presented with the second talker.

### 2.3. Procedure

Participants sat in an enclosed room about 90 cm from a 27-inch computer monitor with two external speakers on either side, located at a 45-degree angle relative to the listener. Participants were given two tasks: the word/pseudoword task, which was split into 2 blocks for each set of stimuli, and the AV McGurk task (1 block). This totaled 3 blocks across the 2 tasks; the order of the tasks was counterbalanced across participants. 

Prior to the start of the Word and Pseudoword blocks, participants were told they would be presented with words, which might sound like real English words or might seem similar to English words. Their task was to listen carefully and type out to the best of their ability exactly what they heard, and not what they lexically thought they heard. These instructions were repeated once again on the monitor prior to the start of the experiment. Participants typed their responses using a keyboard. Stimuli were presented using Presentation v.20.3 (Neurobehavioral Systems, Inc., Berkeley, CA, USA). The Word and Pseudoword blocks consisted of either 39 or 36 trials, respectively, with each stimulus played only once.

For the AV block, prior to starting, participants were informed that they would be presented with videos of an individual producing speech sounds. It was emphasized that participants should always be paying attention to the screen to ensure that they were focused on the talker’s mouth movements. The participants’ task was to type out what they heard. If they heard an ambiguous percept, they were told to transcribe the most dominant percept. These instructions were also presented on the monitor prior to the start of the block. There was a total of 32 trials, with each stimulus repeated twice. An optional two-minute break was offered to participants between each block to mitigate boredom and fatigue.

### 2.4. Data Analysis

Logfiles of participants’ responses were transferred to Excel spreadsheets, which were then parsed using in-house custom MATLAB code (MathWorks, Natick, MA, USA). For the Word and Pseudoword blocks, responses were extracted for each word or pseudoword for each participant. The output of this parsing code was a table containing information about the stimulus, which phoneme was removed in the stimulus, and the response for each trial. An additional column was manually completed, in which we recorded how a participant perceived the removed phoneme on that trial. For example, if the word presented was “addition” with the /ʃ/ (‘sh’ sound) removed and a participant reported perceiving “addithen”, this was coded as ‘th’ filling-in. There were instances where participants perceived no change at all from the original word before a phoneme was removed (e.g., perceiving “addition”) and cases where they reported perceiving the gap itself (e.g., perceiving “addi _on”).

For the AV block, responses were categorized according to the first letter transcribed by the participant (i.e., responses “ba”, “bah”, and “bo” were all included in the response category /b/). The output was a table containing information about the auditory token, visual token, and the first-letter response for each trial.

### 2.5. Statistical Analysis

The data were statistically analyzed in R [24] and MATLAB. Two types of analyses were performed on the data: (1) Mixed effect multinomial logistic regression analyses performed on the auditory-only task data (conducted in R [24]), and (2) a correlation analysis to examine the relationship between ‘/d/t/th/’ perception on the auditory-only task and on the McGurk trials (conducted in MATLAB).

First, we conducted a mixed effects multinomial logistic regression using the mclogit package [25], to examine whether the Block Type (Words vs. Pseudowords) may predict subjects’ auditory perception of the missing phoneme. The outcome measure was the perception of the silent gap (i.e., Response), which comprised four categories: ‘/d/t/th/’ (i.e., filled in the silent gap incorrectly with /d/,/t/, or /th/), Gap (i.e., perceived the silence as a gap without any phonetic filling-in), No Change (i.e., perceived the word or pseudoword by filling in the silent gap with the correct phoneme), and Other Phoneme (i.e., filled in the silent gap with any phoneme except for/d/,/t/,/th/, or the correct phoneme). The /d/t/th/ Response category was set as the referent level, since it was the variable of interest that we wanted to contrast with the other three categories. Importantly, the contrast between /d/t/th/ and Other Phoneme was done to test the hypothesis that when individuals fill-in the silent gap with an incorrect phoneme, they should perceive ‘/d/t/th/’ more often than the other phonemes—especially for the Pseudoword stimuli. 

This initial model included only the fixed effect of Block Type (reference level: Pseudowords), as well as the intercept corresponding to each subject as a random effect. The formula was Response ~ Block Type + 1|SubjectID. An effect of Block Type would reveal that lexical context drives perception of the missing phoneme. This mixed effects multinomial logistic regression model was run using the mblogit function, with the method for modeling the random effects set to the Penalized Quasi-Likelihood (PQL) method. Single-trial data, totaling 2537 trials across all 34 subjects, were inputted into the model with 13 trials (i.e., 0.5% of all 2550 trials) excluded due to missing responses (i.e., the subject pressed “Enter” without typing anything). Relative Risk Ratios (RRR) were computed by exponentiating the coefficients for the fixed effects. In the context of the current analysis, an RRR greater than 1 indicates that missing phonemes in Words are more likely than missing phonemes in Pseudowords to be perceived as Other Phoneme (or Gap or No Change) over ‘/d/t/th/’. An RRR less than 1 indicates the opposite pattern, for example, relative to missing phonemes within Pseudowords, participants were more likely to perceive missing phonemes within Words as ‘/d/t/th/’ than the contrasted perceptual outcome (i.e., Other Phoneme, No Change, or Gap).

We also conducted a secondary mixed-effects multinomial logistic regression analysis, which was an exploratory analysis to assess whether the Manner of Articulation (MoA) of the missing phoneme, as well as its interaction with Block Type, predict auditory perception of the missing phoneme. In this second model, the fixed effects included the Block Type (2 levels: Word or Pseudoword), Manner of Articulation (MoA) of the missing phoneme (3 levels: Fricative/Affricate [ʃ, s, ʒ, z, ʧ, ʤ], Liquid [l, r], and Stop [k, t, d, g, b]), and their interaction, as well as the Syllable from which the phoneme was deleted (2 levels: 2nd or 3rd Syllable). For the fixed effects, the reference levels for the three predictors were the Pseudoword Block Type, the Fricative MoA, and the 2nd Syllable, respectively. Syllable was inputted as a fixed effect to control for any possible syllable effects on perception, since the syllable from which the missing phoneme was removed was not balanced across the MoA categories, as follows: A fricative/affricate was removed from the 2nd syllable of 3 words and 4 pseudowords, and from the 3rd syllable of 20 words and 15 pseudowords. A liquid was removed from the 2nd syllable of 4 words and 4 pseudowords, and from the 3rd syllable of 2 words and 2 pseudowords. A stop consonant was removed from the 2nd syllable of 8 words and 6 pseudowords, and from the 3rd syllable of 2 words and 5 pseudowords. However, the effects of Block Type and MoA, as well as their interaction, were the key effects of interest. The intercept corresponding to each subject was inputted into the model as a random effect. The formula was Response ~ Block Type * MoA + Syllable + 1|SubjectID. To foreshadow the results, follow-up multinomial logistic regression analyses were conducted to interpret significant interaction effects.

Finally, the correlation analysis was done to test the main hypothesis that individuals with stronger auditory-only ‘/d/t/th/’ perception of missing phonemes should exhibit increased ‘/d/t/th/’ perception of the McGurk stimuli. To do this, the total percentage of ‘/d/t/th/’ responses for the auditory-only filling-in trials (collapsed across the type of missing phoneme and word/pseudoword condition) was computed for each subject. Similarly, the total percentage of ‘/d/t/th/’ responses on the McGurk trials was computed for each subject. Subsequently, we conducted Pearson correlations on these two sets of ‘/d/t/th/’ percentages.

## 3. Results

### 3.1. Mixed Effects Multinomial Logistic Regression

**Figure 1:**
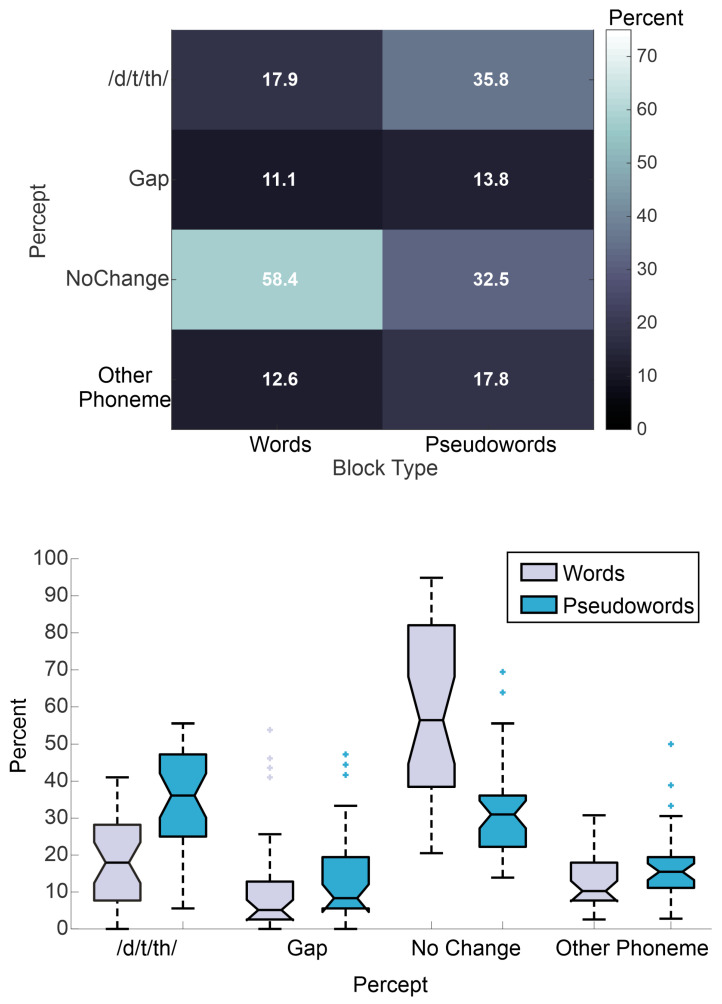
Results of the Auditory-Only Filling-in Task. Top portion shows the group average percentages of each percept, separately for the Word and Pseudoword stimuli. Bottom portion shows box plots of the same data.

The primary multinomial logistic regression analysis was performed to examine how the presence (or absence) of lexico-semantic context affects perception of a missing phoneme in auditory-only stimuli. This was done by inputting Block Type (Words vs. Pseudowords) as a fixed effect into the model. The results are depicted in Table 1 and Figure 1. Recall that ‘/d/t/th/’ perception was set as the referent level for the outcome measure, so that it could be compared with the other three percept categories (Gap, No Change, and Other Phoneme). The relative risk of perceiving the missing phoneme as a Gap vs. ‘/d/t/th/’, No Change vs. ‘/d/t/th/’, and Other Phoneme vs. ‘/d/t/th/’ for Words was significantly higher (i.e., 1.53, 3.86, and 1.41 times higher, respectively), than the same relative risks for Pseudowords. Thus, the presence of lexico-semantic context significantly affected perception, such that participants were most likely to correctly fill-in the missing phoneme for the Word stimuli. On the flip side, missing phonemes within Pseudowords were significantly more likely to be perceived as ‘/d/t/th/’ than both the Gap and Other Phoneme percepts.

**Figure 2:**
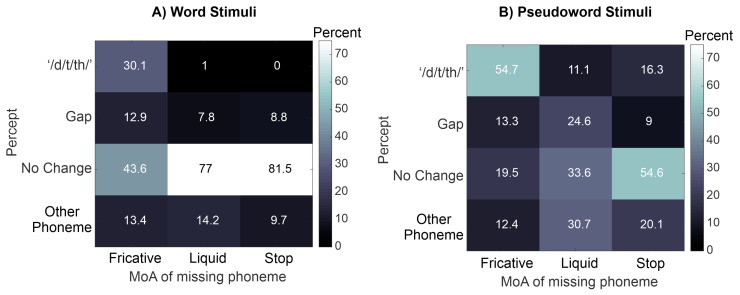
Group-average percentages of each percept reported for the (**A**) Word and (**B**) Pseudoword stimuli, depending on the MoA of the missing phoneme. The percentages of each percept were calculated separately within each MoA category (i.e., each column adds up to 100%).

Upon close examination of the data, it appeared that the missing phoneme’s Manner of Articulation (MoA) may modulate auditory perception. Thus, we also ran a more complex, follow-up multinomial logistic regression as an exploratory analysis to examine whether the Manner of Articulation (MoA) of the missing phoneme and its interaction with Block Type, predict auditory perception of the missing phoneme, while controlling for the Syllable from which the missing phoneme was removed. The results of this mixed effects multinomial regression are presented in Table 2. Additionally, Figure 2 illustrates the percentages that each percept experienced across subjects, within each Block Type (Words, Pseudowords) and MoA.

As shown in Table 2, there was a significant interaction between Block Type and MoA for the No Change vs. ‘/d/t/th/’ contrast. Thus, a follow-up mixed effects multinomial logistic regression was performed to facilitate interpretation of the results. The follow-up analysis was done by re-coding the two predictors, Block Type and MoA, into a single predictor variable, called “BTMoA” which had six levels encoding both the Block Type (Word, Pseudoword) and the Manner of Articulation of the missing phoneme (i.e., Word-Fricative, Word-Stop, Word-Liquid, Pseudoword-Fricative, Pseudoword-Stop, and Pseudoword-Liquid). Like the initial model, the ‘/d/t/th/’ response was set as the referent level for the outcome measure. For the fixed effects, the reference levels included the Pseudoword-Fricative condition and the 2nd Syllable. The intercept corresponding to each subject was inputted into the model as a random effect. The formula for the follow-up model was Response ~ BTMoA + Syllable + 1|SubjectID. An identical follow-up analysis was conducted, but with Word-Fricative as the reference level to directly contrast the effect of MoA within the Word block type.

The results of these follow-up analyses are depicted in Table 3 and Table 4. Please note that a valid estimate could not be generated for the Word*MoA-Stop interaction in the initial multinomial regression and for the Word-Stop condition in these follow-up analyses. Close examination of the data revealed that across the 34 participants, there was never a Word-Stop trial in which the missing phoneme was incorrectly perceived as /d/t/th/; thus, a valid estimate could not be generated. (Word-Stop and Pseudoword-Stop stimuli with a missing /d/ or /t/ that was subsequently perceived as /d/ or /t/, respectively, were categorized as No Change.).

As shown in Table 3, while controlling for the syllable containing the missing phoneme, the relative risks of perceiving Gap vs. /d/t/th/ for the Pseudoword-Liquid, Pseudoword-Stop, Word-Fricative, and Word-Liquid conditions were significantly higher than the same relative risk for the Pseudoword-Fricative condition. The same pattern of results was observed for the No Change vs. /d/t/th/ and Other Phoneme vs. /d/t/th/ contrasts. Specifically, when the Pseudoword-Fricative condition was set as the reference level, the relative risk of perceiving a Gap, No Change, or Other Phoneme vs. /d/t/th/ was 1.81, 4.43, and 2.06 times higher, respectively, for the Word-Fricative condition. Overall, the Pseudoword-Fricative condition was most likely to lead to /d/t/th/ perception compared to the other conditions. As demonstrated in Table 4, while controlling for the syllable with the missing phoneme, the relative risks of perceiving Gap vs. /d/t/th/ for the Pseudoword-Liquid and Word-Liquid conditions were significantly higher than that for the Word-Fricative condition. Moreover, the relative risks of perceiving No Change or Other Phoneme vs. /d/t/th/ for the Pseudoword-Liquid, Pseudoword-Stop, and Word-Liquid conditions were also significantly greater than that for the Word-Fricative condition. 

Taken together, these results suggest that participants were most likely to perceive /d/t/th/ in the place of a missing fricative, and even to a greater extent when the fricative was deleted from a Pseudoword compared to a Word. When the missing phoneme was a liquid, participants were most likely to perceive No Change if the stimulus was a Word, or most likely to perceive a Gap or Other Phoneme if the stimulus was a Pseudoword. Therefore, both the lexical and articulatory context (and their interaction) seem to play a role in shaping listeners’ perception of missing phonemes.

Finally, another incidental finding of this analysis was an effect of Syllable. Specifically, when the missing phoneme was removed from the third syllable, the relative risk of perceiving No Change vs. /d/t/th/ was significantly higher (RRR = 2.69, *p* < 0.001) than when the missing phoneme was removed from the second syllable. There was no effect of Syllable for the Gap vs. /d/t/th/or Other Phoneme vs. /d/t/th/ contrasts. This again suggests an effect of context, such that increasing the amount of preceding context within these trisyllabic words and pseudowords facilitated accurate filling-in of the missing phoneme.

### 3.2. Correlation between Auditory-Only Filling-In and McGurk Illusion

On the audiovisual task, participants performed well on the congruent audiovisual trials, which included congruent /ba/ (96.3% ± 2.1% [mean ± se]), congruent /ga/ (100% ± 0%), and congruent /da/ (89.0% ± 3.5%) stimuli. On the incongruent audiovisual (McGurk) trials comprising /ba/-/ga/ stimuli, there was wide variability in subjects’ susceptibility to the McGurk illusion; 16 of the 34 subjects never experienced the McGurk illusion (i.e., never perceived /d/,/t/, or /th/ on any of the incongruent trials), and 3 of the 34 subjects experienced the illusion on 100% of the incongruent trials.

**Figure 3:**
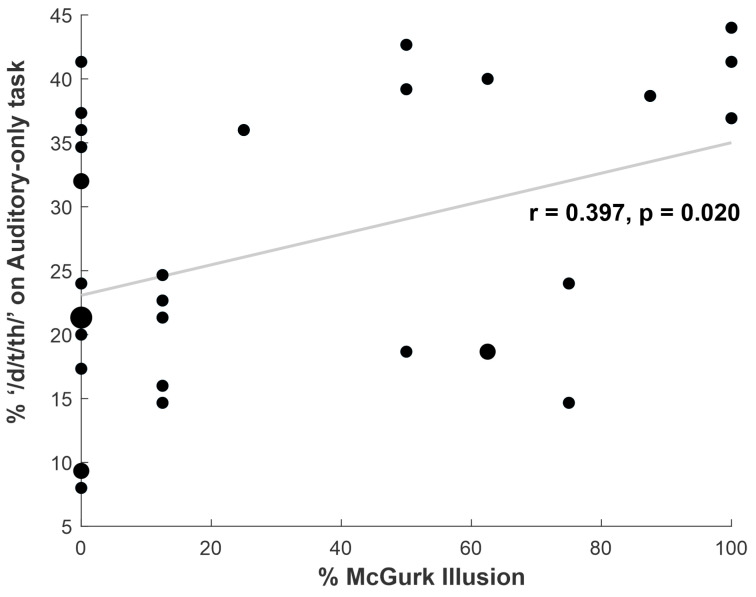
Results of the correlation between /d/t/th/ perception on McGurk trials and on the Auditory-only task. The dots represent individual participants; enlarged dots depict data points shared by two or more participants, with dot size proportional to the number of overlapping participants.

Crucially, as shown in Figure 3, there was a significant across-subjects correlation between the percentage of ‘/d/t/th/’ perception on the auditory-only task (collapsed across word and pseudoword trials) and on the McGurk trials (r = 0.397, *p* = 0.020). Subjects who perceived the McGurk illusion more often also tended to perceive ‘/d/t/th/’ more often on the auditory-only trials. We also conducted follow-up correlations for the word and pseudoword trials separately. The correlation for the pseudoword trials was significant (r = 0.386, *p* = 0.024), and the correlation for the word trials was marginally significant (r = 0.306, *p* = 0.078).

## 4. Discussion

Our results point to an auditory default mechanism whereby AV integration fails due to ambiguity in the visual stimuli, forcing the auditory modality to drive the McGurk illusion on its own (i.e., auditory default). Notably, the results showed that listeners often perceive a gap in auditory-only stimuli as ‘/d/t/th/’—the same percept often perceived during the McGurk illusion. Furthermore, participants who were more likely to perceive ‘/d/t/th/’ on the auditory-only task were also more likely to perceive ‘/d/t/th/’ illusory perception on the McGurk task. 

A remaining challenge is to identify the neuronal basis for this proposed auditory default mechanism. We begin by arguing that current evidence calls into question links between AV illusions and AV integration. We conclude by proposing that the current results support the existence of a default mechanism that favors the auditory modality and thus gives rise to the classic McGurk illusion.

First, assuming parsimony, the same general AV mechanism should underlie the classic McGurk and visual dominance illusions alike, even though they are induced by different pairings of visual and auditory syllables. Just because the stimuli are different, it does not necessarily follow that the AV processing mechanism is distinct. Otherwise, AV processing would be highly inefficient. Second, we know that the general influence of visual modality on auditory modality is suppressive [26,27,28,29,30], a finding further confirmed in our own lab [19]. Third, there is also evidence for a secondary influence of the visual modality on the auditory modality: an encoding phase, in which the visual modality encodes its phonetic representation (viseme) within the auditory modality [17,19,31]. Indeed, in Shahin et al. [19], we demonstrated that visual suppression of the auditory cortex mentioned in the second premise above is deliberate. This cross-modal suppression occurs so that existing auditory representations conveyed by the ear are inhibited to render the auditory modality more prone to alteration by the visual modality (i.e., the cross-modal secondary encoding phase; third premise above). This is necessary because if auditory representations are too robust, it would be difficult for the visual modality to overwrite them. Shahin et al. [19] used the visual dominance illusion to demonstrate this effect: while the N1-P2 auditory evoked potentials were suppressed for AV versus auditory-only conditions; there was a specific encoding effect as well. When individuals heard ‘ba’ when presented with visual-/ba/ and auditory-/fa/, the auditory N1 increased in amplitude (i.e., became more negative). When individuals heard ‘fa’ when presented with visual-/fa/ and auditory-/ba/, the auditory N1 decreased in amplitude. This shift mirrored the relative amplitude difference for /ba/ and /fa/ in the auditory-only condition, with the N1 for /ba/ being larger (more negative) than the N1 for /fa/. 

**Figure 4:**
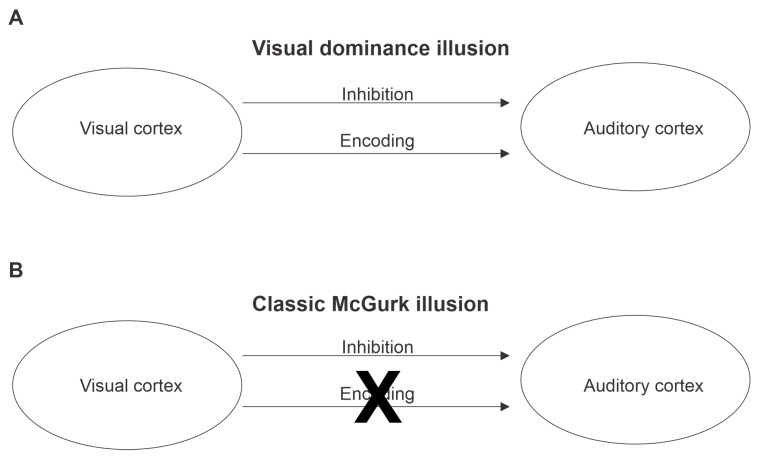
Theoretical Framework illustrating underlying mechanism of the (**A**) Visual dominance illusion and (**B**) Classic McGurk illusion. The Visual Dominance illusion involves visually-mediated inhibition of the auditory cortex, followed by encoding of the visually-conveyed information at the auditory cortex, leading to auditory perception of the visually-conveyed phoneme. According to the proposed “Auditory Default” mechanism underlying the Classic McGurk Illusion, visually-mediated inhibition of the auditory cortex occurs, but because the visual phonetic representation is ambiguous, the visually-mediated encoding step fails, and thus auditory perception is dominated by the phonetic representations with the intrinsically strongest weights within the auditory cortex (‘/d/t/th/’).

Based on the above, we propose a tentative model of the McGurk illusion mechanism. Our theoretical framework illustrated in Figure 4, posits that the classic McGurk illusion follows the same process as the visual dominance illusion, except for one step. Following inhibition of phonetic representations within the auditory modality, the encoding step fails to materialize because the visual utterance of /ga/ or /ka/ is indiscernible—it is confused with /sa/, /ya/, /ha/, /ja/ [9]. Consequently, the auditory system is faced with an ambiguous situation: the auditory input has been inhibited while there is no discernable visual input. As a result, the auditory modality is forced to default to phonetic representations that are naturally dominant (highly weighted) in discourse (i.e., ‘/d/t/th/’). Our framework is consistent with animal work [32,33,34], but deviates from other models, which assert that decisions about multisensory integration occur in higher-level brain regions, such as superior temporal sulcus/gyrus and/or prefrontal cortex [2,35,36,37,38]. These high-level networks evaluate the sum of visual and auditory input and conclude upon a percept [4,39,40].

As for lexical influence, it is interesting that in the auditory-only task, individuals defaulted to ‘d/t/th/’ more often when hearing pseudowords than words. This lexical effect is not surprising given what we know about the phonemic restoration (PR) phenomenon (also known as illusory filling-in). In PR, words with noise-replaced segments can be heard as continuing through the noise (i.e., the speech is perceived as intact) [22,23,41,42,43,44]. The primary difference between the auditory-only task and PR is the difference in replaced segments—silence versus noise. Words exhibit stronger PR than pseudowords, and the more syllables that are within the word, the more robust the PR illusion [43]; both of these effects are consistent with the results from the present auditory-only filling-in task. Moreover, noise is stronger at eliciting PR than silence [45]. However, an interesting result is that insertion of a small silent gap coupled with the noise enhances restoration of stop consonants [44,45,46]. Indeed, even in the present study, relative to pseudowords or words with a missing fricative/affricate, pseudowords with a stop consonant replaced completely by silence were more likely to be accurately filled-in than perceived as ‘/d/t/th/’, and words with a missing stop consonant were never incorrectly perceived as ‘/d/t/th/’. Interestingly, words and pseudowords with missing fricatives/affricates were most often filled-in incorrectly with ‘/d/t/th/’. Together, these results suggest that the lexical and articulatory context modulate the auditory-only filling-in process, but further research is needed to fully understand these incidental findings as they are beyond the scope of the present manuscript. 

There are a few additional issues that warrant our attention. First, recent reports have raised doubts about the suitability of the McGurk illusion as a tool for understanding AV integration of spoken language [11,12]. These researchers assert that the McGurk illusion is based on stimulus manipulations that are rare in real life situations (in particular, see [11]). Such a view is consistent with the reasoning laid out in a recent review of visual-only illusions [47]. However, we do not subscribe to this reasoning. Our view is that manipulating stimuli in ways that rarely—or never—occur in real-life is a powerful way to understand the mechanisms underlying real-life processing. After all, it is our rich history of experiencing situations in real-life that likely gives rise to the experience of illusions. Thus, reverse investigation (“reverse engineering”) is key to understanding the mechanisms at play in ecologically valid situations. Second, because our study suggests that the McGurk illusion is a consequence of failure to integrate AV percepts, it is not surprising that individuals who experience this illusion do not perform better on ecologically valid AV speech comprehension tasks, in line with the conclusions of van Engen et al. [12]. Third, while incongruent AV stimuli in spoken language rarely existed decades ago, we now encounter them often in communication due to video conferencing. In a way, the discovery of the McGurk illusion in 1976, has significantly impacted our understanding of an evolving perceptual phenomenon that is currently often encountered in real life situations—audiovisual incongruency. Thus, the McGurk illusion was well ahead of its time.

## 5. Conclusions

In the current study, we argue that the well-known McGurk illusion may arise due to a failure of audiovisual integration. Consequently, perception is exclusively determined within the auditory modality, such that perception favors (i.e., defaults to) the phonemes often implicated in the McGurk illusion, /d/t/th/. For these reasons, the McGurk illusion is not well suited as a tool to study AV mechanisms in spoken language. However, the McGurk illusion remains an outstanding discovery in language perception research, one that has significantly advanced knowledge in the field.

## Figures and Tables

**Table 1:** Results of the Multinomial Logistic Regression to examine the effect of Block Type (Words vs. Pseudowords) on perception. Significant fixed effects are depicted in bold font and indicated with asterisks as follows: ** *p* < 0.01, *** *p* < 0.001.

**Fixed Effects**						
**Contrast**	**Effect**	**RRR**	**95% CI (LL)**	**95% CI (UL)**	**z**	***p***
**Gap vs. /d/t/th/**	**Intercept**	**0.30**	**0.19**	**0.47**	**−5.21**	<0.001 ***
	Block Type-Pseudoword (ref.)					
	**Block Type-Word**	**1.53**	**1.14**	**2.04**	**2.86**	0.004 **
**No Change vs. /d/t/th/**	Intercept	0.88	0.69	1.11	−1.09	0.276
	Block Type-Pseudoword (ref.)					
	**Block Type-Word**	**3.86**	**3.14**	**4.73**	**12.89**	<0.001 ***
**Other Phoneme vs. /d/t/th/**	**Intercept**	**0.49**	**0.39**	**0.63**	**−5.56**	<0.001 ***
	Block Type-Pseudoword (ref.)					
	**Block Type-Word**	**1.41**	**1.09**	**1.83**	**2.62**	0.009 **
**Random Effects**						
Intercept (Subject ID) Co-variance Parameters	Gap~1		NoChange~1		Other~1	
	Estimate	SE	Estimate	SE	Estimate	SE
Gap~1	1.47	1.21				
No Change~1	0.01	0.08	0.31	0.01		
Other~1	0.26	0.26	0.07	0.02	0.30	0.06

**Table 2:** Results of the Multinomial Logistic Regression to examine the interaction between Block Type and Manner of Articulation on auditory perception (while controlling for the effect of Syllable). Significant fixed effects/interactions are depicted in bold font and indicated with asterisks as follows: ** *p* < 0.01, *** *p* < 0.001.

**Fixed Effects**						
**Contrast**	**Effect**	**RRR**	**95% CI (LL)**	**95% CI (UL)**	**z**	***p***
**Gap vs. /d/t/th/**	**Intercept**	**0.19**	**0.11**	**0.35**	**−5.54**	<0.001 ***
	Block Type-Pseudoword (ref.)					
	**Block Type-Word**	**1.81**	**1.26**	**2.59**	**3.22**	0.001 **
	MoA-Fricative (ref.)					
	**MoA-Liquid**	**11.12**	**6.02**	**20.54**	**7.69**	<0.001 ***
	**MoA-Stop**	**2.12**	**1.24**	**3.65**	**2.73**	0.006 **
	Syllable-2 (ref.)					
	Syllable-3	0.92	0.64	1.32	−0.45	0.654
	Block Type-Word * MoA-Liquid	1.53	0.30	7.71	0.52	0.606
	Block Type-Word * MoA-Stop	-	-	-	-	-
**No Change vs. /d/t/th/**	**Intercept**	**0.14**	**0.09**	**0.21**	**−9.00**	<0.001 ***
	Block Type-Pseudoword (ref.)					
	**Block Type-Word**	**4.43**	**3.35**	**5.87**	**10.42**	<0.001 ***
	MoA-Fricative(ref.)					
	**MoA-Liquid**	**15.66**	**8.89**	**27.60**	**9.52**	<0.001 ***
	**MoA-Stop**	**16.83**	**11.28**	**25.10**	**13.83**	<0.001 ***
	Syllable-2 (ref.)					
	**Syllable-3**	**2.69**	**1.99**	**3.64**	**6.48**	<0.001 ***
	Block Type-Word * MoA-Liquid	**7.39**	**1.63**	**33.51**	**2.60**	0.009 **
	Block Type-Word * MoA-Stop	-	-	-	-	-
**Other Phoneme vs. /d/t/th/**	**Intercept**	**0.20**	**0.13**	**0.30**	**−7.73**	<0.001 ***
	Block Type-Pseudoword (ref.)					
	**Block Type-Word**	**2.06**	**1.46**	**2.90**	**4.15**	<0.001 ***
	MoA-Fricative(ref.)					
	**MoA-Liquid**	**15.00**	**8.42**	**26.71**	**9.20**	<0.001 ***
	**MoA-Stop**	**6.35**	**4.05**	**9.95**	**8.06**	<0.001 ***
	Syllable-2 (ref.)					
	Syllable-3	1.16	0.84	1.62	0.91	0.364
	Block Type-Word * MoA-Liquid	2.50	0.53	11.83	1.15	0.249
	Block Type-Word * MoA-Stop	-	-	-	-	-
**Random Effects**						
Intercept (Subject ID) Co-variance Parameters	Gap ~1		No Change ~1		Other ~1	
	Estimate	SE	Estimate	SE	Estimate	SE
Gap ~1	1.72	2.63				
No Change ~1	0.20	0.65	0.63	0.19		
Other ~1	0.37	0.76	0.22	0.20	0.38	0.23

**Table 3:** Results of the follow-up mixed effects multinomial logistic regression, with Pseudoword-Fricative as the reference category for the Block Type-Manner of Articulation condition. Significant fixed effects/interactions are depicted in bold font and indicated with asterisks as follows: ** *p* < 0.01, *** *p* < 0.001.

**Fixed Effects**						
**Contrast**	**Effect**	**RRR**	**95% CI (LL)**	**95% CI (UL)**	**z**	***p***
**Gap vs. /d/t/th/**	**Intercept**	**0.19**	**0.11**	**0.35**	**−5.56**	<0.001 ***
	Pseudoword-Fricative (ref.)					
	**Pseudoword-Liquid**	**11.10**	**6.01**	**20.51**	**7.69**	<0.001 ***
	**Pseudoword-Stop**	**2.12**	**1.23**	**3.64**	**2.72**	0.006 **
	**Word-Fricative**	**1.81**	**1.26**	**2.59**	**3.22**	0.001 **
	**Word-Liquid**	**30.69**	**6.69**	**140.67**	**4.41**	<0.001 ***
	Word-Stop	-	-	-	-	-
	Syllable-2 (ref.)					
	Syllable-3	0.92	0.64	1.32	−0.45	0.653
**No Change vs. /d/t/th/**	**Intercept**	**0.14**	**0.09**	**0.21**	**−9.01**	<0.001 ***
	Pseudoword-Fricative (ref.)					
	**Pseudoword-Liquid**	**15.65**	**8.88**	**27.58**	**9.51**	<0.001 ***
	**Pseudoword-Stop**	**16.82**	**11.27**	**25.09**	**13.83**	<0.001 ***
	**Word-Fricative**	**4.43**	**3.35**	**5.87**	**10.42**	<0.001 ***
	**Word-Liquid**	**513.19**	**122.58**	**2148.45**	**8.54**	<0.001 ***
	Word-Stop	-	-	-	-	-
	Syllable-2 (ref.)					
	**Syllable-3**	**2.69**	**2.00**	**3.63**	**6.45**	<0.001 ***
**Other Phoneme vs. /d/t/th/**	**Intercept**	**0.20**	**0.13**	**0.30**	**−7.74**	<0.001 ***
	Pseudoword-Fricative(ref.)					
	**Pseudoword-Liquid**	**14.99**	**8.42**	**26.69**	**9.20**	<0.001 ***
	**Pseudoword-Stop**	**6.35**	**4.05**	**9.95**	**8.06**	<0.001 ***
	**Word-Fricative**	**2.06**	**1.46**	**2.90**	**4.15**	<0.001 ***
	**Word-Liquid**	**77.24**	**17.76**	**335.97**	**5.80**	<0.001 ***
	Word-Stop	-	-	-	-	-
	Syllable-2 (ref.)					
	Syllable-3	1.16	0.84	1.62	0.91	0.364
**Random Effects**						
Intercept (Subject ID) Co-variance Parameters	Gap ~1		No Change ~1		Other ~1	
	Estimate	SE	Estimate	SE	Estimate	SE
Gap ~1	1.69	2.44				
No Change ~1	0.19	0.59	0.63	0.18		
Other ~1	0.36	0.69	0.22	0.18	0.38	0.21

**Table 4:** Results of the follow-up mixed effects multinomial logistic regression, with Word-Fricative as the reference category for the Block Type-Manner of Articulation condition. Please note that the random effects covariance parameters are not displayed, since they are identical to Table 3. Significant fixed effects/interactions are depicted in bold font and indicated with asterisks as follows: * *p* < 0.05, ** *p* < 0.01, *** *p* < 0.001.

**Fixed Effects**						
**Contrast**	**Effect**	**RRR**	**95% CI (LL)**	**95% CI (UL)**	**z**	***p***
**Gap vs. /d/t/th/**	**Intercept**	**0.35**	**0.19**	**0.63**	**−3.50**	<0.001 ***
	Word-Fricative(ref.)					
	**Pseudoword-Fricative**	**0.55**	**0.39**	**0.79**	**−3.21**	0.001 **
	**Pseudoword-Liquid**	**6.15**	**3.32**	**11.40**	**5.76**	<0.001 ***
	Pseudoword-Stop	1.17	0.68	2.02	0.58	0.563
	**Word-Liquid**	**17.00**	**3.70**	**78.01**	**3.64**	<0.001 ***
	Word-Stop	-	-	-	-	-
	Syllable-2 (ref.)					
	Syllable-3	0.92	0.64	1.32	−0.45	0.653
**No Change vs. /d/t/th/**	**Intercept**	**0.61**	**0.40**	**0.93**	**−2.32**	0.020 *
	Word-Fricative(ref.)					
	**Pseudoword-Fricative**	**0.23**	**0.17**	**0.30**	**−10.42**	<0.001 ***
	**Pseudoword-Liquid**	**3.53**	**2.03**	**6.14**	**4.47**	<0.001 ***
	**Pseudoword-Stop**	**3.79**	**2.60**	**5.54**	**6.90**	<0.001 ***
	**Word-Liquid**	**115.76**	**27.83**	**481.56**	**6.53**	<0.001 ***
	Word-Stop	-	-	-	-	-
	Syllable-2 (ref.)					
	**Syllable-3**	**2.69**	**1.99**	**3.64**	**6.45**	<0.001 ***
**Other Phoneme vs. /d/t/th/**	**Intercept**	**0.40**	**0.26**	**0.61**	**−4.27**	<0.001 ***
	Word- Fricative(ref.)					
	**Pseudoword-Fricative**	**0.23**	**0.17**	**0.30**	**−4.14**	<0.001 ***
	**Pseudoword-Liquid**	**7.27**	**4.09**	**12.93**	**6.76**	<0.001 ***
	**Pseudoword-Stop**	**3.08**	**1.97**	**4.81**	**4.94**	<0.001 ***
	**Word-Liquid**	**37.47**	**8.62**	**162.87**	**4.83**	<0.001 ***
	Word-Stop	-	-	-	-	-
	Syllable-2 (ref.)					
	Syllable-3	1.16	0.84	1.62	0.91	0.364

## Data Availability

To access the data collected from this experiment, please click on the link to the Figshare site: https://figshare.com/projects/The_McGurk_illusion_A_default_mechanism_of_the_auditory_system/156281 (accessed on 2 February 2023).

## References

[1] McGurk H., MacDonald J. (1976). Hearing Lips and Seeing Voices. Nature.

[2] Beauchamp M.S., Lee K.E., Argall B.D., Martin A. (2004). Integration of Auditory and Visual Information about Objects in Superior Temporal Sulcus. Neuron.

[3] Beauchamp M.S., Nath A.R., Pasalar S. (2010). FMRI-Guided Transcranial Magnetic Stimulation Reveals That the Superior Temporal Sulcus Is a Cortical Locus of the McGurk Effect. J. Neurosci..

[4] Erickson L.C., Zielinski B.A., Zielinski J.E.V., Liu G., Turkeltaub P.E., Leaver A.M., Rauschecker J.P. (2014). Distinct Cortical Locations for Integration of Audiovisual Speech and the McGurk Effect. Front. Psychol..

[5] Tremblay C., Champoux F., Voss P., Bacon B.A., Lepore F., Théoret H. (2007). Speech and Non-Speech Audio-Visual Illusions: A Developmental Study. PLoS ONE.

[6] Hirst R.J., Stacey J.E., Cragg L., Stacey P.C., Allen H.A. (2018). The Threshold for the McGurk Effect in Audio-Visual Noise Decreases with Development. Sci. Rep..

[7] Sekiyama K., Soshi T., Sakamoto S. (2014). Enhanced Audiovisual Integration with Aging in Speech Perception: A Heightened McGurk Effect in Older Adults. Front. Psychol..

[8] Pearl D., Yodashkin-Porat D., Katz N., Valevski A., Aizenberg D., Sigler M., Weizman A., Kikinzon L. (2009). Differences in Audiovisual Integration, as Measured by McGurk Phenomenon, among Adult and Adolescent Patients with Schizophrenia and Age-Matched Healthy Control Groups. Compr. Psychiatry.

[9] Gonzales M.G., Backer K.C., Mandujano B., Shahin A.J. (2021). Rethinking the Mechanisms Underlying the McGurk Illusion. Front. Hum. Neurosci..

[10] Getz L.M., Toscano J.C. (2021). Rethinking the McGurk Effect as a Perceptual Illusion. Atten. Percept. Psychophys..

[11] Van Engen K.J., Dey A., Sommers M.S., Peelle J.E. (2022). Audiovisual Speech Perception: Moving beyond McGurk. J. Acoust. Soc. Am..

[12] Van Engen K.J., Xie Z., Chandrasekaran B. (2017). Audiovisual Sentence Recognition Not Predicted by Susceptibility to the McGurk Effect. Atten. Percept. Psychophys..

[13] Grant K.W., Seitz P.-F. (2000). The Use of Visible Speech Cues for Improving Auditory Detection of Spoken Sentences. J. Acoust. Soc. Am..

[14] Schorr E.A., Fox N.A., van Wassenhove V., Knudsen E.I. (2005). Auditory-Visual Fusion in Speech Perception in Children with Cochlear Implants. Proc. Natl. Acad. Sci. USA.

[15] Sumby W.H., Pollack I. (1954). Visual Contribution to Speech Intelligibility in Noise. J. Acoust. Soc. Am..

[16] Rosenblum L.D., Saldaña H.M. (1992). Discrimination Tests of Visually Influenced Syllables. Percept. Psychophys..

[17] Abbott N.T., Shahin A.J. (2018). Cross-Modal Phonetic Encoding Facilitates the McGurk Illusion and Phonemic Restoration. J. Neurophysiol..

[18] Alsius A., Paré M., Munhall K.G. (2018). Forty Years After Hearing Lips and Seeing Voices: The McGurk Effect Revisited. Multisens. Res..

[19] Shahin A.J., Backer K.C., Rosenblum L.D., Kerlin J.R. (2018). Neural Mechanisms Underlying Cross-Modal Phonetic Encoding. J. Neurosci..

[20] Miller G.A., Nicely P.E. (1955). An Analysis of Perceptual Confusions Among Some English Consonants. J. Acoust. Soc. Am..

[21] Anderson J.L., Morgan J.L., White K.S. (2003). A Statistical Basis for Speech Sound Discrimination. Lang. Speech.

[22] Shahin A.J., Miller L.M. (2009). Multisensory Integrati3on Enhances Phonemic Restoration. J. Acoust. Soc. Am..

[23] Shahin A.J., Bishop C.W., Miller L.M. (2009). Neural Mechanisms for Illusory Filling-in of Degraded Speech. NeuroImage.

[24] R Core Team (2019). R: A Language and Environment for Statistical Computing. https://www.R-project.org/.

[25] Elff M. (2021). mclogit: Multinomial Logit Models, with or without Random Effects or Overdispersion. https://CRAN.R-project.org/package=mclogit.

[26] Besle J., Fort A., Delpuech C., Giard M.-H. (2004). Bimodal Speech: Early Suppressive Visual Effects in Human Auditory Cortex. Eur. J. Neurosci..

[27] van Wassenhove V., Grant K.W., Poeppel D. (2005). Visual Speech Speeds up the Neural Processing of Auditory Speech. Proc. Natl. Acad. Sci. USA.

[28] Stekelenburg J.J., Vroomen J. (2007). Neural Correlates of Multisensory Integration of Ecologically Valid Audiovisual Events. J. Cogn. Neurosci..

[29] Pilling M. (2009). Auditory Event-Related Potentials (ERPs) in Audiovisual Speech Perception. J. Speech Lang. Hear. Res..

[30] Shatzer H., Shen S., Kerlin J.R., Pitt M.A., Shahin A.J. (2018). Neurophysiology Underlying Influence of Stimulus Reliability on Audiovisual Integration. Eur. J. Neurosci..

[31] Smith E., Duede S., Hanrahan S., Davis T., House P., Greger B. (2013). Seeing Is Believing: Neural Representations of Visual Stimuli in Human Auditory Cortex Correlate with Illusory Auditory Perceptions. PLoS ONE.

[32] Ghazanfar A.A. (2005). Multisensory Integration of Dynamic Faces and Voices in Rhesus Monkey Auditory Cortex. J. Neurosci..

[33] Kayser C., Petkov C.I., Logothetis N.K. (2008). Visual Modulation of Neurons in Auditory Cortex. Cerebral Cortex.

[34] Kayser C., Logothetis N.K., Panzeri S. (2010). Visual Enhancement of the Information Representation in Auditory Cortex. Curr. Biol..

[35] Calvert G.A., Campbell R., Brammer M.J. (2000). Evidence from Functional Magnetic Resonance Imaging of Crossmodal Binding in the Human Heteromodal Cortex. Curr. Biol..

[36] Noppeney U., Ostwald D., Werner S. (2010). Perceptual Decisions Formed by Accumulation of Audiovisual Evidence in Prefrontal Cortex. J. Neurosci..

[37] Romanski L.M. (2012). Convergence of Auditory, Visual, and Somatosensory Information in Ventral Prefrontal Cortex. The Neural Bases of Multisensory Processes.

[38] Hwang J., Romanski L.M. (2015). Prefrontal Neuronal Responses during Audiovisual Mnemonic Processing. J. Neurosci..

[39] Miller L.M., D’Esposito M. (2005). Perceptual Fusion and Stimulus Coincidence in the Cross-Modal Integration of Speech. J. Neurosci..

[40] Morís Fernández L., Macaluso E., Soto-Faraco S. (2017). Audiovisual Integration as Conflict Resolution: The Conflict of the McGurk Illusion: The Conflict of the McGurk Illusion. Hum. Brain Mapp..

[41] Warren R.M. (1970). Perceptual Restoration of Missing Speech Sounds. Science.

[42] Warren R.M., Hainsworth K.R., Brubaker B.S., Bashford J.A., Healy E.W. (1997). Spectral Restoration of Speech: Intelligibility Is Increased by Inserting Noise in Spectral Gaps. Percept. Psychophys..

[43] Samuel A.G. (1981). Phonemic Restoration: Insights from a New Methodology. J. Exp. Psychol. Gen..

[44] Samuel A.G. (1981). The Role of Bottom-up Confirmation in the Phonemic Restoration Illusion. J. Exp. Psychol. Hum. Percept. Perform..

[45] Warren R.M., Obusek C.J. (1971). Speech Perception and Phonemic Restorations. Percept. Psychophys..

[46] Sherman G. (1971). The Phonemic Restoration Effect: An Insight into the Mechanisms of Speech Perception. Unpublished Master’s Thesis.

[47] Rogers B. (2022). When Is an Illusion Not an Illusion? An Alternative View of the Illusion Concept. Front. Hum. Neurosci..

